# Bilateral congenital severe microphthalmia in two siblings

**DOI:** 10.11604/pamj.2022.43.69.35059

**Published:** 2022-10-11

**Authors:** Jihene Sayadi, Zeineb Kallel

**Affiliations:** 1 Department “A”, Hedi Rais Institute of Ophthalmology, Tunis El-Manar University, Tunis, Tunisia

**Keywords:** Microphthalmia, anophthalmia, congenital abnormality

## Image in medicine

Microphthalmia, anophthalmia, and coloboma, fall under the MAC spectrum of ocular malformations. Congenital anophthalmia/microphthalmia is a rare developmental condition. It is due to the almost complete defect of the primary optic vesicle, which leads to an absent or very small eye within the orbit. The birth prevalence of these abnormalities is up to 3 per 10,000 births. The diagnosis is established upon clinical and imaging criteria. Genetic counselling might be challenging due to the wide range of involved genes. A full-term male newborn was referred to our department on the first day of life for ophthalmic examination. Medical history revealed a parental consanguinity. The patient was the third child for both parents. They had a six-year-old daughter with isolated bilateral severe microphthalmia (A,B) and a 4-year-old healthy son. Prenatal genetic analysis of both parents and the old daughter did not identify chromosomal abnormalities. Additionally, gestational-acquired infections, maternal vitamin A deficiency, X-rays or drugs exposure were excluded. Upon examination of the newborn, we noticed a firmly closed eyes. The eyelid shape was normal. A gentle pose of the speculum revealed narrowed palpebral fissures and small conjunctival fornices. Both eyeballs, especially the left one, had a very reduced size. Conjunctiva and cornea were barely individualized. Adnexal tissues including the lacrimal canal were identified (C). Further systemic evaluation including transfontanellar and abdominal ultrasound, as well as neurological and urological assessments ruled out associated anomalies. Computed tomography scan of the orbits confirmed the diagnosis of severe bilateral microphthalmia. Rudimentary optic nerves were detected. Extra ocular muscles were within normal limits (D).

**Figure 1:**
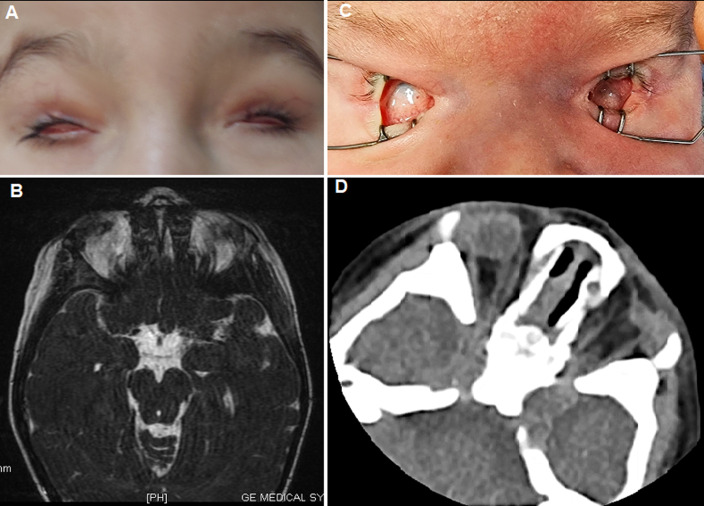
A) eye photograph of the six-year-old sister showing bilateral severe microphthalmia; B) T2-weighted MR scan of the sister revealing rudimentary severely hypoplastic eyeballs and the presence of amorphous tissue and structures; C) clinical appearance of the bilateral severe microphthalmia in the newborn; D) coronal FLAIR sequence of the newborn demonstrating rudimentary severely hypoplastic eyeballs and vestigial optic nerves

